# Managing Medical Emergencies in Hungarian Pharmacies

**DOI:** 10.3390/pharmacy7030095

**Published:** 2019-07-17

**Authors:** Peter P. Felkai, Zsolt Iván

**Affiliations:** Faculty of Pharmacology, Semmelweis University, 1085 Budapest, Hungary

**Keywords:** emergencies, pharmacy, first aid, pharmacists’ attitude, vaccination, postgraduate education, children

## Abstract

To amass a body of knowledge for managing emergency situations in pharmacies, we surveyed the occurrence and nature of medical problems in Hungarian pharmacies. The occurrence of real or suspected emergencies in pharmacies was markedly different and varied from 1–52 per year, with five cases per year on average. The most frequent problems were bleeding (69%) and dizziness (55%), but other more serious problems (allergic reaction (32%), collapse (23%), and chest pain (25%)) also occurred. Sometimes more than one symptom was reported by a patient. People appear to consider pharmacies to be an appropriate site for receiving first aid for minor ailments, including common medical problems (e.g., fever (12%)). Unfortunately, the range of interventions was very limited because of local legal regulations and the lack of appropriate guidelines for emergencies in pharmacies. The most frequent interventions were wound treatment, control of bleeding (78%), alleviation of anxiety (68%), and providing patients with a glass of water (55%). Very often, more than one intervention was reported for the same case. Whereas 76.3% of pharmacists provided interventions only for adults, 21% of pharmacists provided interventions for all types of patients (adults, co-workers in pharmacies, and children). Pharmacists appeared to be reluctant to treat children, owing to the special issues related to pediatrics. This poor range of intervention should encourage responsible officials to develop guidelines for pharmacists to ensure pharmacists’ familiarity with the appropriate interventions in emergency situations. Such knowledge could also provide a good basis for preparing pharmacists to perform vaccinations in the future. The pharmacists had a positive attitude toward providing first aid, and 88% of respondents requested more postgraduate education on medical first aid issues.

## 1. Introduction

Immunization plays a key role in public health programs in all countries, with the goal of preventing communicable diseases. The administration of vaccines is performed by health-care professionals, most frequently nurses, according to national immunization schedules. In Hungary, the mandatory vaccination program is fairly comprehensive and is performed exclusively by pediatricians (childhood vaccination), and the seasonal immunizations (that are free of charge) are performed by general practitioners. Pre-travel immunization is performed in the outpatient clinics of health-care facilities and by some private travel clinics, and travelers are required to pay all charges (such as vaccines and pre-travel counseling). Vaccinations should be performed exclusively by doctors. Travelers are most frequently vaccinated by their general practitioner [1] for a small fee.

There is currently a shortage of physicians in Hungary, and this has resulted in consequences such as overworked staff, difficulties in obtaining appointments, and long waiting times in the clinic; these consequences might make people reluctant to obtain the necessary pre-travel vaccinations. In addition to this problem, some travelers are reluctant to obtain the necessary vaccinations because of unfounded fears related to adverse effects, e.g., autism. Consequently, immunization uptake is low, and thousands of unused seasonal vaccines are lost.

One solution would be to increase access to vaccinations through the pharmacy system, as is already a growing practice in many countries [2,3]. Pharmacists in many countries have the appropriate motivation and ability to perform immunizations [4]. Moreover, the opening times of most pharmacies would also contribute to providing better access. Pharmacists could play a crucial role in vaccination counseling [5], thereby increasing vaccine uptake [6]. 

However, some professionals and officials have concerns regarding pharmacists’ lack of necessary training, skills, and appropriate knowledge in the field of advanced life support, which are crucial in managing the adverse effects that could occur during vaccination. The certification program for pharmacists has been well established in postgraduate education programs in many countries [7].

To introduce this new activity in pharmacies in Hungary, pharmacists must first have the postgraduate education and training, and must maintain the knowledge and skills necessary to provide first aid response, life support, and management of casualties and incidents until the arrival of medical assistance or an ambulance. 

The main aim of this study was to obtain data on Hungarian pharmacists’ attitudes, knowledge, and everyday practice in emergencies occurring in their offices. The obtained information should allow the relevant committee to establish a body of knowledge regarding the topics and to compile national guidelines and training plans for Hungarian pharmacists.

## 2. Methods

A questionnaire was uploaded to an internet site for pharmacists. The 22 questions in the questionnaire were related to emergency situations or medical problems that had occurred in their respective pharmacies. The term “emergency situation” referred to any sudden deterioration in a patient’s health status that required first aid intervention, basic life support, or even an ambulance call. The questions focused on medical problems that had already occurred in the given pharmacies, notably symptoms that could evolve into medical emergencies and the treatment of these symptoms, including through the provided first aid methods.

One hundred and thirteen pharmacists completed the questionnaire; 98% of the respondents worked in community pharmacies, and 2% worked in hospital pharmacies. One response per pharmacy was selected. The anonymity of the respondents was carefully maintained. Almost all age groups were represented, as displayed in Table 1.

The respondents’ qualifications were as follows: pharmacist [Master of Pharmacy degree] (75%); senior pharmacist [specialized postgraduate diploma] (21%); or pharmacy technician (4%) (Table 2) (All responders were considered “pharmacists” in this study. The pharmacy technicians in Hungary are entitled to provide more services and independent activities for clients than in other countries. That is why they can perform first-aid as well). Among the respondents, 84.1% were female, and 15.9% were male. 

The answers were analyzed in IBM–SPSS Statistics for Windows, Version 24.0 (IBM Corp 2016) statistical software. Although only 5% of all Hungarian pharmacies responded to the questionnaire, we consider this survey a preliminary study and emphasize that more advanced and detailed research is needed for a proper full-scale analysis.

In Hungary, there are approximately 2010 community pharmacies and 143 hospital pharmacies. Despite the low number of respondents (<10% of all pharmacies in Hungary) and the unknown response rate, some relevant findings were obtained, and several conclusions were made. We hope that this preliminary study will provide valuable data on emergency interventions (mainly basic first aid) in pharmacies; however, further study is needed to obtain more precise data on emergencies that frequently occur in pharmacies.

## 3. Results

### 3.1. Incidence of Emergency Situations

All respondents had observed one or more alarming situations in their offices. A total of 574 emergency (or emergency-like) situations were reported in the pharmacies. The average number of emergencies was five cases per pharmacy per year. The observed events ranged from 1–52 emergencies per pharmacy (Table 3). 

The reported frequency of emergencies in the pharmacies is shown in Table 4.

### 3.2. Medical Problems Occurring in Pharmacies

The nature of the medical problems and their treatment in pharmacies were also evaluated. Medical problems (one or more) that occurred in the pharmacies were as follows (Table 5):

### 3.3. Interventions Provided

**Figure 1:**
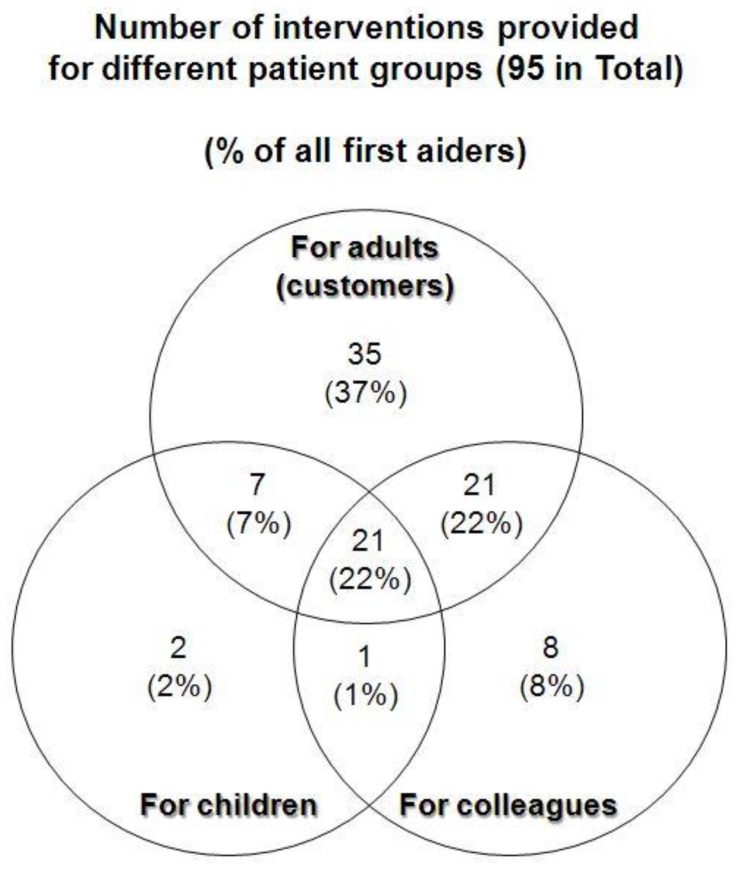
Number of interventions provided for different patient groups (95 in total).

Among 113 respondents, 95 provided first aid or took action to help the patients in their pharmacies. Three groups of patients required interventions in pharmacies: adults, colleagues in pharmacies, and children. Among the 95 pharmacists, 2% treated children only, and 37% treated adults only (Figure 1). Only 21% of pharmacists treated all groups of patients.

The total reported number of interventions was 524. The types of intervention are shown in Table 6. When the relatively narrow scale of interventions was examined, Hungarian pharmacists’ limited legal competency to perform medical activity should be kept in mind. In emergencies, all respondents reported one or more interventions.

### 3.4. The Final Section of the Questionnaire Was Related to the Pharmacists’ Attitudes Toward Postgraduate Education in Medical First Aid

Almost all pharmacists (88%) indicated that they would like to improve their knowledge in the field of professional first aid. The most requested topics of postgraduate courses were first aid for injuries (such as bleeding, minor wounds, and head and spine injuries), recognition of the origins of different illnesses that could result in syncope, and treating panic disorders.

**Figure 2:**
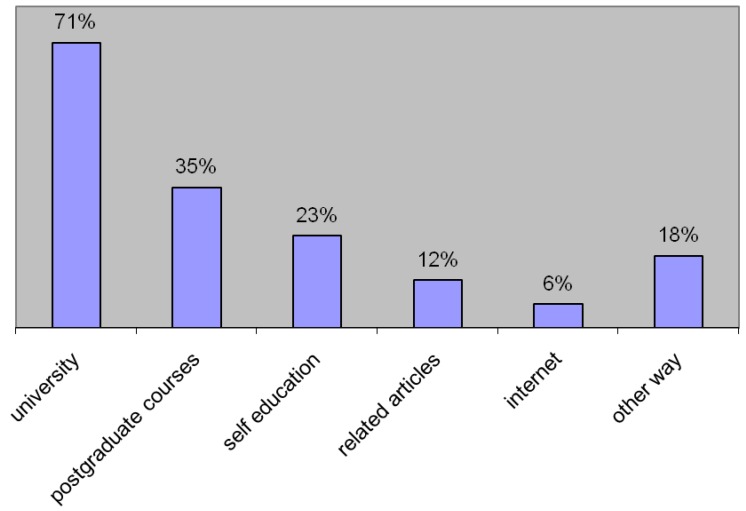
The sources of medical intervention knowledge (more than one answer was also accepted).

In relation to their present knowledge in the field of first aid, the pharmacists mentioned the sources of their knowledge (Figure 2).

## 4. Discussion

The occurrence of emergencies (or emergency-like situations) in pharmacies was extremely varied and ranged from 1–52 cases per year, with five cases per month on average. These findings suggest that emergencies can occur frequently. When the background information from the questionnaire was analyzed, no correlation was observed between the age or gender of the first aid/intervention provider, or the size of the community in which the pharmacies operate. Almost all respondents had been involved in an emergency (or emergency-like) situation in pharmacies, and most pharmacists had carried out therapeutic interventions.

Although pharmacies are usually visited by ill people, people with sudden medical problems also believe that any pharmacist can provide first aid. Thus, pharmacists may play a vital role in providing medical help [8]. This conclusion was drawn on the basis of the majority of minor ailments treated, such as bleeding and burns. The most frequent medical problem was bleeding (generally due to injury or nose bleeds) which accounted for 69% of the total number of medical problems. The second most frequent medical problem was dizziness (55%), which can be an alarming symptom and may require immediate help from the pharmacist. The relatively large percentage of allergic reactions (32%) was striking, because this symptom can originate from medicines taken in pharmacies or shortly after patients leave pharmacies. Symptoms such as collapse and chest pain may indicate more serious problems, and postgraduate courses on Advanced Life Support (ALS) and a better understanding of the mechanism of syncope, collapse, and acute coronary symptoms, and the nature of different medical emergencies, would be beneficial for pharmacists. All other mentioned medical problems were common disorders such as fever, abdominal pain, and minor trauma.

The limited legal regulations related to therapy administered by pharmacists, and sometimes the lack of medical tools and supplies, make pharmacists’ therapeutic efforts limited and unvarying. Control of bleeding was the most frequent therapy applied. Some pharmacists mentioned that they alleviated patients’ anxiety as a therapy. Providing patients with a glass of water was a frequent routine (or “do-something” phenomena?) procedure. The interventions in most cases were no more than low-level first aid interventions. Owing to the obligatory cardiopulmonary resuscitation (CPR) lessons during university education, the initiation of CPR was also performed by pharmacists. According to the analysis shown in Figure 1, all patient groups (adult, co-workers, and children) were treated by pharmacists. High motivation for providing interventions was found for the adult group (customers and co-workers), but significantly fewer pharmacists attempted to help children. Among 113 respondents, 95 provided first aid or attempted to help, and had a strong motivation to provide professional help. The current poor therapeutic activities might possibly have been a reason for the high demand for postgraduate education in various fields.

Most (88%) respondents requested more postgraduate courses on basic life support and ALS. The positive attitude of pharmacists toward postgraduate courses is well documented in the literature [9]. We found that 71% of respondents acquired their knowledge on first aid and CPR during university lectures. Sometimes the duration between university lectures and knowledge utilization in everyday practice was too long, and the knowledge had faded. Although emergency situations are relatively frequent in pharmacies, training is crucial in maintaining skills. Treating most of the reported emergency situations (bleeding, collapse, and allergic reactions) requires up-to-date information and, consequently, regular postgraduate education. The lack of postgraduate education is also an issue in other countries [10]. Impressively, 25% of respondents obtained knowledge on first aid through self-education.

Suggestions from pharmacists for future postgraduate topics included the differential diagnosis of panic disorder, the appropriate way to summon an ambulance, and the treatment of asthma in children. These suggestions were passed on to the authors of the guidelines. A textbook on these topics (including Basic Life Support (BLS) for children) had already been published in 2016 [11].

If regulations allow, and Hungarian pharmacists are eager to provide vaccinations and pre-travel advice, the possibility of providing travel-related vaccinations will be achievable, and the optimal position of community pharmacists in providing pre-travel consultation will be properly utilized [12]. However, pharmacists must take part in further education programs ranging from basic first aid interventions to good practice in ALS treatment. Knowledge on basic life support and advanced first aid protocols must be outlined, because many countries have this type of regulation [7]. Hopefully, this preliminary study may provide support in developing this role.

## Figures and Tables

**Table 1:** Age distribution of the respondents.

Age Group (Years)	% of Respondents
20–30	15%
31–40	35%
41–50	17%
>50	33%

**Table 2:** Distribution of respondents by professional level.

Educational Level	No. of Respondents	Percent
Pharmacist	85	75.2
Senior Pharmacist	24	21.2
Pharmacy technicians	4	3.6
Total	113	100.0

**Table 3:** Average yearly incidence rate of emergencies in the pharmacies.

Average Number of Emergencies/Year	Number of Pharmacy Reported Incidences within This Range	Percent (Rounded)
0–4	76	67
5–9	25	22
10–19	8	7
≥ 20	4	4

**Table 4:** Frequency of emergency or emergency-like medical problems.

Frequency of Emergencies	Number of Pharmacy Reported This Frequency	Percent (Rounded)
once a week	4	4
monthly	8	7
rarely	101	89

**Table 5:** Medical problems occurring in the pharmacy (one respondent could mention more than one problem).

Symptoms	Mentioned Medical Problems by % of Pharmacists
Bleeding (injury)	69%
Dizziness (dazed walk)	55%
Burns, scalds	34%
Allergic reaction	32%
Collapse	29%
Chest pain	25%
Dyspnea, short of breath	19%
Injury of an extremity (sprain, strain, fracture)	18%
Abdominal pain, cramp	16%
Fever (chill, shivering)	12%
Others, miscellaneous	13%

**Table 6:** Interventions provided by pharmacists.

Intervention	Applied by Pharmacists %
Bandaging, control bleeding	78%
Alleviating anxiety	68%
Provide a glass of water	55%
Place the patient in the recumbent position	45%
Initiate cardiopulmonary resuscitation	1%
Other interventions, not specified	5%
